# Development of elliptic core-shell nanoparticles with fluorinated surfactants for ^19^F MRI

**DOI:** 10.3389/fchem.2024.1408509

**Published:** 2024-06-12

**Authors:** Yue Wu, Masafumi Minoshima, Kazuya Kikuchi

**Affiliations:** ^1^ Graduate School of Engineering, Osaka University, Suita, Japan; ^2^ Japan Science and Technology Agency PRESTO, Suita, Japan; ^3^ Immunology Frontier Research Center, Osaka University, Suita, Japan

**Keywords:** ^19^F MRI, fluorinated surfactant, nanoparticles, silica nanoparticles, core-shell nanoparticles

## Abstract

Perfluorocarbon-encapsulated silica nanoparticles possess attractive features such as biological inertness and favorable colloidal properties for bioimaging with fluorine magnetic resonance imaging (^19^F MRI). Herein, a series of elliptic shaped silica nanoparticles with perfluorocarbon liquid perfluoro-15-crown-5 ether as core (PFCE@SiO_2_) were synthesized using fluorinated surfactants *N*-(perfluorononylmethyl)-*N*,*N*,*N*-trimethylammonium chloride (C10-TAC) and *N*-(perfluoroheptylmethyl)-*N*,*N*,*N*-trimethylammonium chloride (C8-TAC). The nanoparticles are characterized to obtain elliptic core-shell structures. PFCE@SiO_2_ showed strong ^19^F NMR signals of the encapsulated PFCE, indicating the potential as a highly sensitive ^19^F MRI probe. These elliptic PFCE@SiO_2_ nanoparticles provide a new option of ^19^F MRI probe with a morphology different from conventional nanospheres.

## 1 Introduction

Magnetic resonance imaging (MRI) is one of the most promising imaging techniques benefiting to its deep tissue penetration, high special resolution, non-invasiveness, and non-radioactivity. In conventional proton (^1^H) MRI, intrinsic proton such as water and fat results in relatively high background signal that could not be ignored even under the circumstance of using contrast agent, which brings imaging contrast issue (Louie et al., 2000). To compensate this issue, a strategy of “hot spot” imaging using heteronuclear ^19^F has come into sight. Apart from the favorable NMR properties of ^19^F, since only trace amounts of ^19^F are present in the human body, fluorine MRI contrast agent can be detected without background signal (Ahrens et al., 2005). However, the sensitivity issue of ^19^F MRI caused by the limited dosage of F atoms remains a major problem of ^19^F MRI.

Perfluorocarbon nano-emulsion is one of the most widely utilized currently developed ^19^F MRI probes (Tirotta et al., 2015). They are consisted of nanosized liquid perfluorocarbon droplets stabilized by a lipid monolayer. Nano-emulsion droplets typically have a particle size in the range of 100–200 nm. Perfluorocarbon nano-emulsion can encapsulate a large amount of perfluorocarbon molecules in liquid state and thus have improved MR sensitivity. One example is perfluoro (Yamabe et al., 2000) crown-5-ether (PFCE) nanoemulsions. PFCE has various attractive characteristics as a^19^F MRI contrast agent as it has 20 equivalent fluorine atoms, which show a sharp single ^19^F NMR peak to yield a strong signal. PFCE nanoemulsions have been reported for *in vivo* cell tracking (Matsushita et al., 2014). However, the stability of PFCE nano-emulsion is limited by flocculation, coalescence, and Ostwald ripening (Flögel and Ahrens, 2017). This significantly limits their application through surface modifications.

To improve the properties of PFCE nano-emulsion, we have developed a novel multifunctional ^19^F MRI contrast agent, named fluorine accumulated silica nanoparticle for MRI contrast enhancement (FLAME) (Matsushita et al., 2014). FLAME is a nanoparticle that has a core-shell structure similar with PFCE nano-emulsion where liquid perfluoro-15-crown-5 ether (PFCE) is encapsulated in silica shell. The 20 chemical equivalent fluorine atoms in PFCE molecule bring FLAME high MR sensitivity. Benefiting from the silica coating, flocculation, coalescence, and Ostwald ripening could be prevented. Thus, FLAME has been proved to have high stability and high surface modifiability. Through adequate surface modifications of FLAME, various applications such as protein labeling, cell labeling, and tumor targeting were achieved. In addition, by changing core compound of FLAME nanoparticles, multicolor ^19^F MRI were also achieved.

In recent years, there has been increased attention on the biodistribution and activity of nanomaterials. Many attempts have been made for controlling the morphologies and properties of various types of nanomaterials (Kladko et al., 2021). We have modified the surface functional groups (Akazawa et al., 2018), area (Nakamura et al., 2015), and elasticity (Konishi et al., 2023) of the perfluorocarbon encapsulated nanoparticles for regulating their biological effects. Despite these efforts, controlling of the size and shape in such perfluorocarbon encapsulated nanoparticles remains challenging.

To address this limitation, we focus on fluorinated surfactants as emulsifier during the preparation of the nanoparticles. Fluorinated surfactants have unique behaviors compared to classical surfactants. Fluorine atoms in these surfactants exhibit very low surface tension in aqueous solution even when used at reduced concentrations (Pabon and Corpart, 2002). This property leads to the stabilization of perfluorocarbon nanoemulstions (Astafyeva et al., 2015). Fluorinated surfactants can behave as an alternative surfactant for uniform and size-controlled synthesis of silica nanoparticles in conventional methods. Moreover, the size, shape, and dispersibility of the particles can also be tuned by using fluorinated surfactants (Nagappan et al., 2021).

Herein, we prepared perfluorocarbon encapsulated silica nanoparticles using two kinds of cationic fluorinated surfactants with different alkyl chain lengths to investigate the influences of the size and shape. We found the formation of elliptic core-shell nanoparticles with 100–200 nm size by using *N*-(perfluorononylmethyl)-*N*,*N*,*N*-trimethylammonium chloride (C10-TAC) or *N*-(perfluoroheptylmethyl)-*N*,*N*,*N*-trimethylammonium chloride (C8-TAC) as a surfactant. Furthermore, the elliptic nanoparticles exhibited strong ^19^F NMR signals derived from the perfluorocarbon molecules in the core, which is suitable as ^19^F MRI probes. These elliptic PFCE@SiO_2_ nanoparticles provide a new option of ^19^F MRI probe with a novel morphology that is different from general nanospheres.

## 2 Materials and methods

### 2.1 Materials

Best grade general reagents were supplied by Tokyo Chemical Industry Co., Ltd. (Tokyo, Japan), Watanabe Chemical Co., Ltd. (Hiroshima, Japan), FUJIFILM Wako Pure Chemical Corporation (Osaka, Japan), Sigma-Aldrich Co. LLC (St. Louis, MO, United States), and NOF CORPORATION (Tokyo, Japan). Elastic carbon-coated copper grid was bought from Okenshoji Co., Ltd. for TEM measurement. (Tokyo, Japan).

### 2.2 Synthesis of C10-TAC and C8-TAC

C10-TAC and C8-TAC were synthesized according to the procedures in the previous report (Matsuoka et al., 2007). The synthetic schemes are shown in Supplementary Figures 1, 2.

#### 2.2.1 *N*-(perfluorononylmethyl) -*N*,*N*,*N*-trimethylammonium chloride (C10-TAC)

Dimethylamine methanol solution (10%, 15 mL) was titrated to methyl perfluorodecanoate (**1a**) (2.50 g, 5.13 mmol) dispersed in 5 mL of methanol and was stirred for 24 h. The solution was dried by vacuum evaporation to produce *N*,*N*-dimethyl-perfluorodecanamide as a yellow solid (**2a**) (100%). The solid dissolved in diethyl ether was titrated into LiAlH_4_ (1.25 g, 32.94 mmol) dispersed in 50 mL of diethyl ether at −10°C. After 3 days of stirring at room temperature, water (1.25 mL), 15% NaOH aqueous solution (1.25 mL), and water (3.75 mL) were added slowly and stepwise to the diethyl solution cooled by iced water. The white solid was filtered and washed with fresh diethyl ether. The diethyl ether solution was dried with anhydrous Na_2_SO_4_ and then evaporated to afford 1.04 g of N-(1,1-Dihydroperfluorodecyl)-*N*,*N*-dimethylamine (**3a**, 42.6%). ^1^H NMR (CDCl_3_): 3.00 (CH_2_, t, *J* = 16 Hz, 2H), 2.44 (CH_3_, s, 6H).

Methyl iodide (1.63 g, 11.48 mmol) was titrated to **3a** (1.04 g, 1.97 mmol), dissolved in ethanol (5 mL) at room temperature, and refluxed in an oil bath for 4 h. After cooling to room temperature, the solution was poured into 6 mL of diethyl ether. The solution with a white solid was stored at −20°C for 1 night, and the solid was filtered to afford *N*-(perfluorononylmethyl)-*N,N,N*-trimethylammonium iodide **4a** (49%). After drying in a vacuum, the solid was dissolved in 100 mL of methanol, stirred with the ion-exchange resin for 2 h, and recrystallized with 1:1 methanol: cyclohexane, to afford 396.1 mg (26.3 mmol) of *N*-(perfluorononylmethyl)-*N*,*N*,*N*-trimethylammonium chloride (C10-TAC) (63.0%). ^1^H NMR (CD_3_OD): 4.67 (CH_2_, t, *J* = 16 Hz, 2H), 3.47 (CH_3_, s, 9H).

#### 2.2.2 *N*-(perfluoroheptylmethyl) -*N*,*N*,*N*-trimethylammonium chloride (C8-TAC)

Dimethylamine methanol solution (10%, 35 mL) was titrated to methyl perfluorooctanoate (**1b**) (5.00 g, 11.68 mmol) dispersed in 5 mL of methanol and was stirred for 24 h. The solution was dried by vacuum evaporation to produce *N*,*N*-dimethyl-perfluorooctanamide as a yellow solid (**2b**) (96.7%). The solid dissolved in diethyl ether was titrated into LiAlH_4_ (3.07 g, 80 mmol) dispersed in 10 mL of diethyl ether at −10°C. After 3 days of stirring at room temperature, water (2.5 mL), 15% NaOH aqueous solution (2.5 mL), and water (7.5 mL) were added slowly and stepwise to the diethyl solution cooled by iced water. The white solid was filtered and washed with fresh diethyl ether. The diethyl ether solution was dried with anhydrous Na_2_SO_4_ and then evaporated to afford 2.67 g of *N*-(1,1-Dihydroperfluorooctyl)-*N*,*N*-dimethylamine (**3b**) (55.7%). ^1^H NMR (CDCl_3_): 3.10 (CH_2_, t, *J* = 16.5 Hz, 2H), 2.41 (CH_3_, s, 6H).

Methyl iodide (4.84 g, 34.12 mmol) was titrated to **3b** (2.50 g, 5.85 mmol), dissolved in ethanol (10 mL) at room temperature, and refluxed in an oil bath for 4 h. After cooling to room temperature, the solution was poured into 12 mL of diethyl ether. The solution with a white solid was stored at −20°C for 1 night, and the solid was filtered to afford *N*-(perfluoroheptylmethyl)-*N,N,N*-trimethylammonium iodide **4b** (1.97 g, 59.0%). After drying in a vacuum, the solid was dissolved in 40 mL of methanol, stirred with the ion-exchange resin for 2 h, and recrystallized with 1:1 methanol: acetone, to afford 656.6 mg of *N*-(perfluoroheptylmethyl)-*N*,*N*,*N*-trimethylammonium chloride (C8-TAC) (56.1%). ^1^H NMR (CD_3_OD): 4.63 (CH_2_, t, *J* = 16.5 Hz, 2H), 3.44 (CH_3_, s, 9H).

### 2.3 Synthesis of elliptic core-shell nanoparticles using fluorinated surfactants

C10-TAC (4.26 mg) or C8-TAC (25.98 mg) and *N*-(2-(2-(pyridin-4-yl)acetamido)ethyl) palmitamide (PAP) (0.33 mg) were dissolved in 5 mL of water using a bath-type sonicator (Branson 1250). Then 35 µL of PFCE was added to the emulsion, followed by sonication by a bath-type sonicator (sonifire) for 120 min at 65°C. The emulsion was filtrated with 0.45 µm filter (Millipore, hydrophilic PFPE). Water (10 mL) and TMOS (tetramethyl orthosilicate) (100 µL) was added to the emulsion, and then the mixture was stirred for 72 h at room temperature. The product was purified by centrifugation (15,000 rpm, 5°C, 30 min), washed three times with ethyl alcohol. Purified FLAME-OH was dispersed with water.

### 2.4 Characterization of nanoparticles

NMR spectra were recorded on Bruker AscendTM 500 instrument (Billerica, MA, United States) at 470 MHz. The ^19^F NMR spectra were referenced to TFANa (sodium trifluoroacetate). The PFCE concentration was measured using ^19^F NMR by mixing 450 μL of the sample with 45 μL of D_2_O and 5 μL of 100 mM TFANa. The long transverse relaxation time (*T*_2_) was measured using the spin-echo method. Hydrodynamic radius was measured by dynamic light scattering (DLS) at 25°C with a 580 nm laser at a scattering angle of 90° for size measurements. For the size measurements, nanoparticles were suspended in H_2_O. Transmission electron microscopy (TEM) images were acquired by using a JEOL JEM-1400 (Tokyo, Japan) at 100 kV.

### 2.5 Stability of nanoparticles

Nanoparticles with a PFCE concentration of 0.8 mM were dispersed in Hanks’ Balanced Salts Solution (HBSS) buffer at pH = 7.4. The sample was stored at room temperature. The hydrodynamic radius, PFCE concentration and *T*_2_ were measured by dynamic light scattering (DLS) and NMR respectively at indicated time points.

### 2.6 Cytotoxicity assay

HeLa cells were seeded in 96-well plates with a concentration of 1 × 10^4^/well and incubated for 24 h in DMEM (containing 10% FBS). After washed twice with HBSS buffer, the media were replaced by HBSS containing indicated concentrations of nanoparticles, and the cells were incubated for 2 h. Then, the cells were washed 3 times by HBSS and incubated with DMEM containing WST-8 (Dojindo, Japan) for another 1 h. The absorbance at 450 nm of the medium was measured with a microplate reader ARVO mx (PerkinElmer, CT, United States).

## 3 Results

### 3.1 Design and synthesis of fluorinated surfactant C10-TAC and C8-TAC

For preparation of silica nanoparticles containing perfluorocarbon core, two types of fluorinated surfactants *N*-(perfluorononylmethyl)-*N*,*N*,*N*-trimethylammonium chloride (C10-TAC) and *N*-(perfluoroheptylmethyl)-*N*,*N*,*N*-trimethylammonium chloride (C8-TAC) were chosen according to their aggregated structure (Matsuoka et al., 2007). C10-TAC forms two types of aggregates, disc-shaped lamellas with diameters less than 30 nm and unilamellar vesicle with hydrodynamic diameter of approximately 100 nm above their critical micelle concentration (CMC). The aggregates of C8-TAC are formed in a poly-disperse state, with larger aggregates at approximately 10–30 nm and smaller ones less than ca. 5 nm, above CMC (Matsuoka et al., 2007). In contrast, According to the previous report, C8-TAC and C10-TAC form normal spherical micelles, while surfactants with perfluorocarbon chains longer than C8- and C10-TAC such as C12-TAC form unilamellar structures with a diameter of 100 nm (Matsuoka et al., 2007). On the other hand, surfactants with a shorter perfluorocarbon chain such as C6-TAC is likely to be in an extended state at the surface of the micelles and will be in closer contact with water. This results in lower aggregation efficiency and a higher CMC, which is not really ideal choice for surfactant (Wang et al., 2005). We assumed that surfactants that forms normal spherical micelles can more efficiently stabilize perfluorocarbons. Therefore, only C8-TAC and C10-TAC were chosen as candidates for nanoparticles preparation.

**FIGURE 1:**
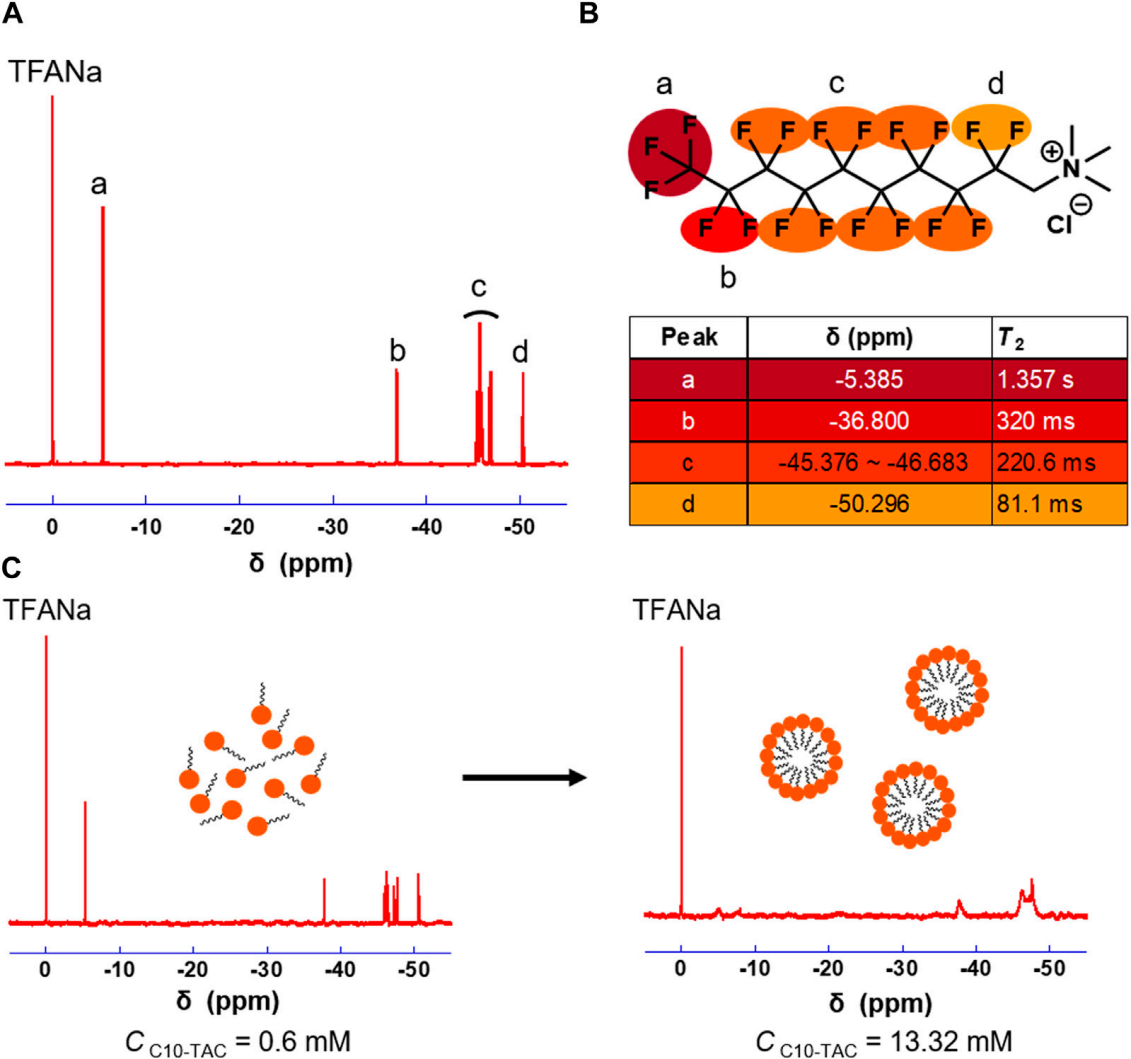
^19^F NMR properties of C10-TAC. **(A)** ^19^F NMR of C10-TAC. **(B)** *T*_2_ of corresponding fluorine atoms. **(C)** ^19^F NMR changes under aggregation of C10-TAC (left: C10-TAC = 0.6 mM, right: C10-TAC = 13.32 mM).

C10-TAC and C8-TAC were synthesized according to previous report (Supplementary Figures S1, S2) (Yamabe et al., 2000). ^19^F NMR of C10-TAC were measured in methanol (Figure 1A). The transverse relaxation time (*T*_2_) value of respective peak in C10-TAC were then investigated to evaluate the mobility of each fluorine atoms (Figure 1B). *T*_2_ value decreased in the order from the fluorous tail (a position) to the hydrophilic head (d position), indicating the reduction of mobility of fluorine atoms at the head position even in the polar solvent. The aggregation behaviors of C10-TAC were then investigated using ^19^F NMR at concentrations above and below its CMC (1.28 mM) in water (Matsuoka et al., 2007) (Figure 1C). When C10-TAC was measured at 0.48 mM below CMC (Figure 1C, left), the NMR peaks were sharp. In contrast, at 13.32 mM above CMC (Figure 1C, right), the NMR peaks were broadened, especially at the tail position. This indicates that when C10-TAC forms micelles, the mobility of fluorine atoms on the alkyl chain is suppressed.

### 3.2 Preparation of nanoparticles

**FIGURE 2:**
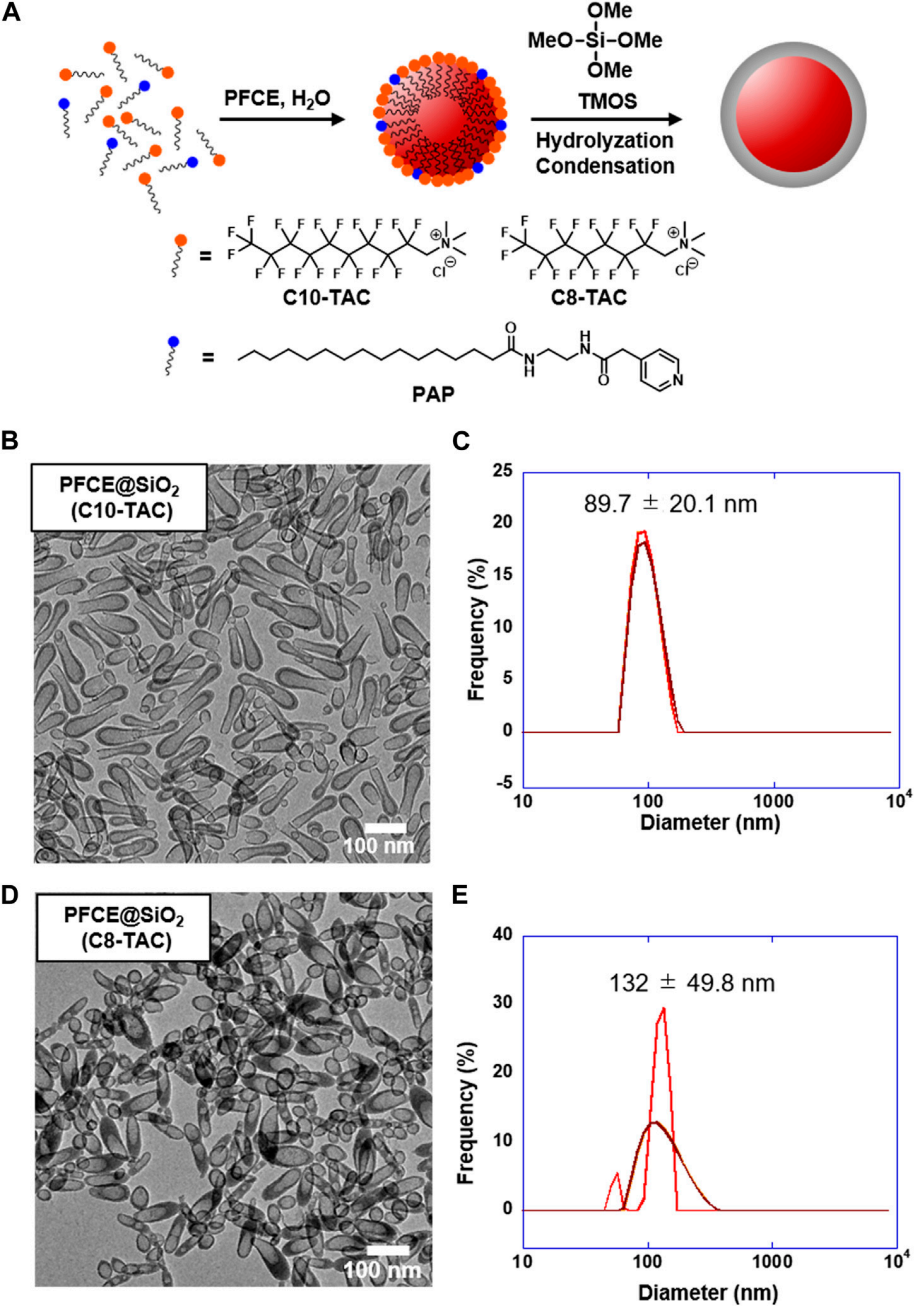
Design and synthesis of PFCE@SiO_2_ by C10-TAC and C8-TAC. **(A)** Preparation scheme of PFCE@SiO_2_. **(B)** Transmission electron microscopy (TEM) image of PFCE@SiO_2_-C10-TAC (Scale bar: 100 nm). **(C)** Hydrodynamic radius of PFCE@SiO_2_-C10-TAC measured by Dynamic light scattering (DLS). **(D)** TEM image of PFCE@SiO_2_-C8-TAC (Scale bar: 100 nm). **(E)** Hydrodynamic radius of PFCE@SiO_2_-C8-TAC measured by DLS.

The synthetic protocol of PFCE@SiO_2_ is presented in Figure 2A. The protocol was modified according to the previous paper (Matsushita et al., 2014). To cover the PFCE nanoemulsion with silica, our group has developed a novel surfactant, *N*-(2-(2-(pyridin-4-yl)acetamido)ethyl) palmitamide (PAP), which has a basic pyridinyl group that can be displayed on the nanoemulsion surface during nanoemulsion formation. We then initiated the sol–gel process of silica precursor tetramethyl orthosilicate (TMOS) on the surface of the nanoemulsion. In the case of preparing nanoparticles with fluorinated surfactant C10-TAC or C8-TAC, the concentration of surfactant was calculated from CMC and fixed at 1 × CMC (Nagappan et al., 2021). The surfactant was mixed with PAP with optimized ratio of 0.33 mg for the best formation of nanoemulsion (Supplementary Table S1; Supplementary Figure S3).

### 3.3 Characterization of nanoparticles

The transmission electron microscopy (TEM) image of obtained PFCE@SiO_2_-C10-TAC is shown in Figure 2B; Supplementary Figure S4A. The TEM image indicates rod-like, elliptic nanoparticles with incomplete core-shell structures. The size of the nanoparticles was approximately 50 × 150 nm. However, the hydrodynamic radius of the nanoparticles was measured by dynamic light scattering (DLS) and determined as 89.7 ± 20.1 nm (Figure 2C). The difference is likely due to the mixture of incompletely formed smaller nanoparticles. On the other hand, the TEM image of PFCE@SiO_2_-C8-TAC is shown in Figure 2D; Supplementary Figure S4B, indicating a core-shell formation of elliptic nanoparticles with a size of approximately 50 × 120 nm. The hydrodynamic radius of nanoparticles was 132 ± 49.8 nm (Figure 2E).

### 3.4 ^19^F NMR properties

**FIGURE 3:**
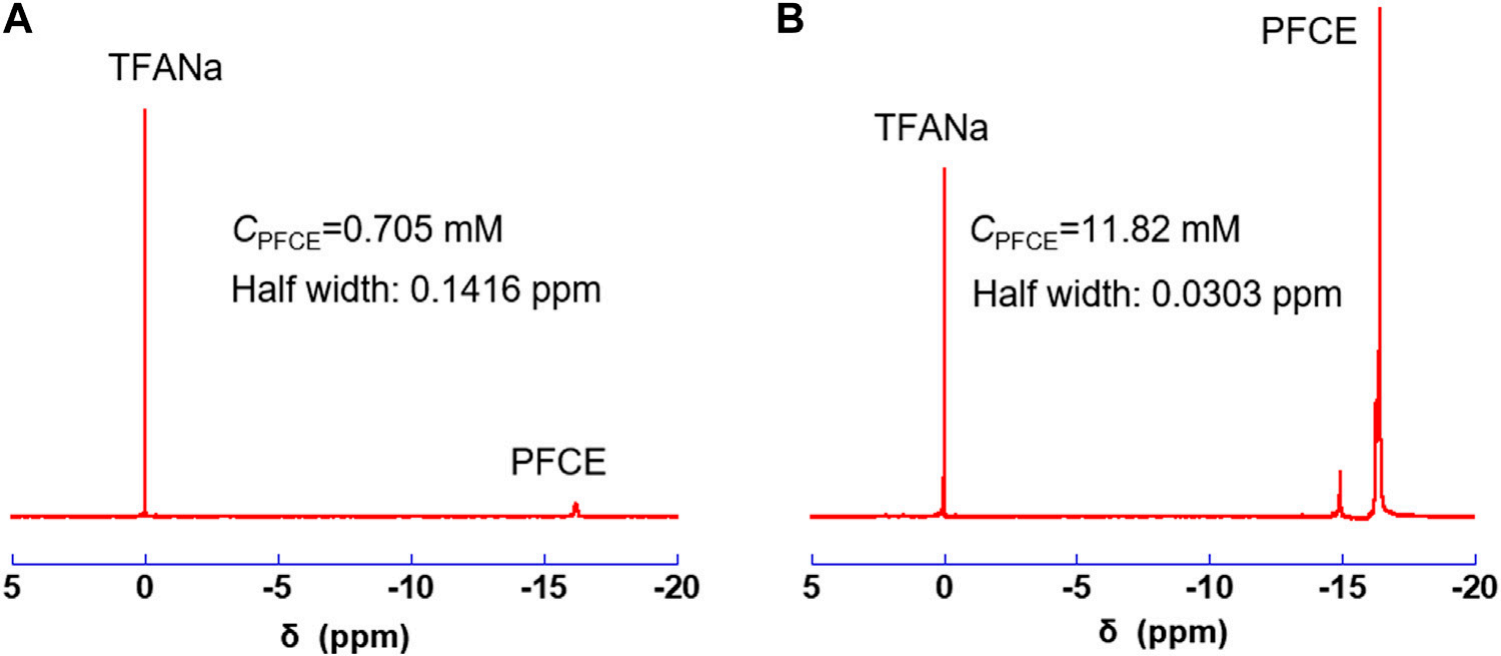
^19^F NMR of PFCE@SiO_2_-C10-TAC **(A)** and PFCE@SiO_2_-C8-TAC **(B)**.

^19^F NMR spectra of PFCE@SiO_2_-C10-TAC and PFCE@SiO_2_-C8-TAC are shown in Figures 3A, B respectively. The PFCE concentration (*C*_PFCE_) in each nanoparticle was determined using TFANa as an internal standard. The *C*_PFCE_ of PFCE@SiO_2_-C10-TAC was 0.705 mM. Meanwhile, the *C*_PFCE_ in PFCE@SiO_2_-C8-TAC nanoparticles is 11.82 mM, which is significantly higher than that of PFCE@SiO_2_-C10-TAC. In addition, the *T*_2_ of PFCE encapsulated in PFCE@SiO_2_-C8-TAC was 346 ms, which is sufficient for ^19^F MRI. The difference is because of the particle formation of PFCE@SiO_2_-C10-TAC and PFCE@SiO_2_-C8-TAC as shown in Figures 2B, D. PFCE@SiO_2_-C10-TAC was longer morphology and had an incomplete core-shell formation, this may cause unsuccessful encapsulation of PFCE and severe loss during purification with centrifugation. In contrast, PFCE@SiO_2_-C8-TAC forms complete core-shell structure, possessing high encapsulation rate of PFCE.

## 4 Discussion

Perfluorocarbon encapsulated nanoemulsions and nanoparticles are promising materials as a contrast agent using ^19^F MRI. In particular, the silica-coated nanoparticles containing perfluorocarbon core improve the stability in aqueous solution, enabling *in vivo* ^19^F MRI. The core-shell silica nanoparticles are currently prepared by forming nanoemulsions containing perfluorocarbon and subsequently polymerizing silica on the emulsion surface. While the surface properties of the silica nanoparticles can be controlled by facile surface modifications, the size and shape control of the nanoparticles highly depends on the initial process of the nanoemulsion formation. This eventually produces sphere-shaped core-shell nanoparticles. In this study, perfluorocarbon encapsulated core-shell nanoparticles were prepared using cationic perfluorinated surfactants, C8-TAC and C10-TAC. Using these surfactants, the elliptic nanoparticles with core-shell structures were obtained (Figures 2B, D), which is significantly different from the sphere nanoparticles using its hydrogenated analogues of the surfactant (Vallet-Regí et al., 2022). This morphological change can be explained by the rigid structure of the single fluorocarbon chain in tetraalkylammonium-based amphiphiles, which leads to the formation of nanomicelles with less-curved lamellar structure (Giulieri and Krafft, 1994; Matsuoka et al., 2006). The aggregated structures also depend on the number of fluoroalkyl chain, which corresponded with the rod-like structure bearing the larger aspect ratio of PFCE@SiO_2_-C10-TAC than PFCE@SiO_2_-C8-TAC.

Comparing with the two fluorinated surfactants, C8-TAC afforded elliptic silica nanoparticles with core-shell structures that contains a number of PFCE molecules. This results in sharp ^19^F NMR signals of the PFCE core (Figure 3B). In contrast, C10-TAC afforded incomplete SiO_2_-shell formation and encapsulation of PFCE molecules, resulting in low signals of the core (Figure 3A). Although the difference in the formation of core-shell particles is currently unclear, it may be attributed to the shape or stability between C10-TAC and C8-TAC micelles. The PFCE@SiO_2_-C8-TAC nanoparticles showed strong ^19^F NMR signals of PFCE core without significant decrease of *T*_2_ value. The relatively high *T*_2_ value indicates the encapsulation of PFCE molecules as a liquid state, as mentioned in our previous spherical silica nanoparticles (Matsushita et al., 2014). The ^19^F NMR properties of PFCE@SiO_2_-C8-TAC are suitable for detection of the perfluorocarbon signals with ^19^F MRI. Furthermore, PFCE@SiO_2_-C8-TAC is stable under physiological condition (Supplementary Figure S5), and do not show significant acute cytotoxicity (Supplementary Figure S6), which makes PFCE@SiO_2_-C8-TAC a promising candidate for *in vivo* ^19^F MRI.

In conclusion, we developed perfluorocarbon encapsulated elliptic core-shell nanoparticles using fluorinated alkylammonium surfactants. The nanoparticles PFCE@SiO_2_-C8-TAC exhibited strong and sharp ^19^F NMR signals, demonstrating encapsulation of core PFCE molecules without the loss of molecular mobility. Our perfluorocarbon encapsulated core-shell nanoparticles can measure the number of nanoparticles taken up by cells using ^19^F NMR. In addition, the localization in tissues can be monitored in real-time with ^19^F MRI without the background signals (Nakamura et al., 2015). The shape of the nanoparticle is one of the important factors for cellular uptake and biodistribution (Vallet-Regí et al., 2022). For example, previous report has demonstrated that rod-shaped mesoporous silica nanoparticles (MSNs) have higher cellular uptake upon A375 cell line compared with sphere shaped MSNs with same radius (Huang et al., 2010). The elliptic nanoparticles will be used as a probe for evaluating the biological destiny of the nanomaterials in living cells and animals.

## Data Availability

The original contributions presented in the study are included in the article/Supplementary Material, further inquiries can be directed to the corresponding author.
