# Supplementary material for: Development of elliptic core-shell nanoparticles with fluorinated surfactants for 19F MRI
Source: Front Chem. 2024 Jun 12;12:1408509. doi: 10.3389/fchem.2024.1408509 (PMC11199681; doi:10.3389/fchem.2024.1408509)
Supplement: Supplementary file 1 [file DataSheet1.docx]

Supplementary Material

**Supplementary Figure 1.** Synthetic scheme of *N*-(perfluorononylmethyl)-*N*,*N*,*N*-trimethylammonium chloride (C10-TAC).

**Supplementary Figure 2.** Synthetic scheme of *N*-(perfluoroheptylmethyl)-*N*,*N*,*N*-trimethylammonium chloride (C8-TAC).

| **Entry** | 1 | 2 | 3 | 4 |
| --- | --- | --- | --- | --- |
| **C10-TAC (mg)** | 4.26 | 4.26 | 4.26 | 8.52 |
| **PAP (mg)** | 0.33 | 0.66 | 0.99 | 0.66 |
| **PFCE (μL)** | 35 | 35 | 35 | 35 |

**Supplementary Table 1.** Amount of reagents of nanoemulsion preparation using *N*-(perfluorononylmethyl)-*N*,*N*,*N*-trimethylammonium chloride (C10-TAC).


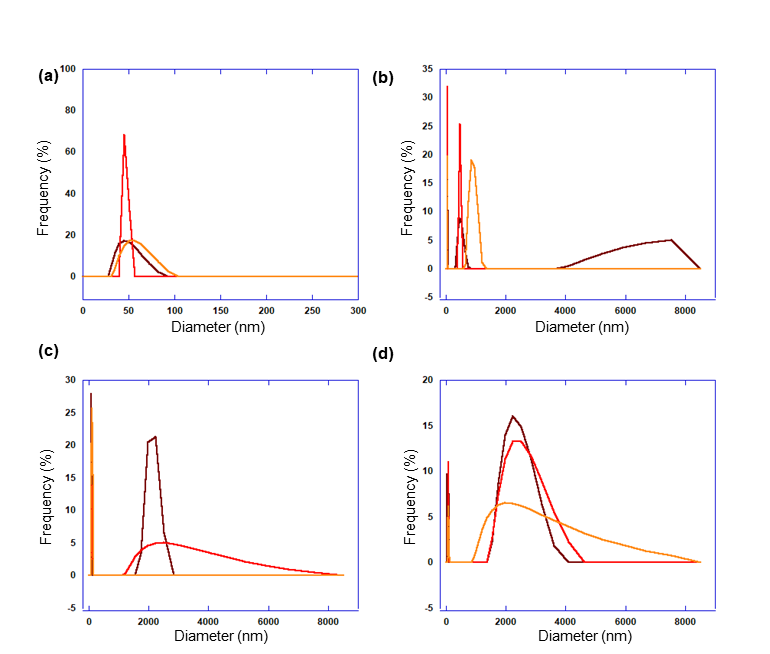


**Supplementary Figure 3.** Hydrodynamic radius of the nanoemulsions using *N*-(perfluorononylmethyl)-*N*,*N*,*N*-trimethylammonium chloride (C10-TAC) in entry 1-4 (a-d).


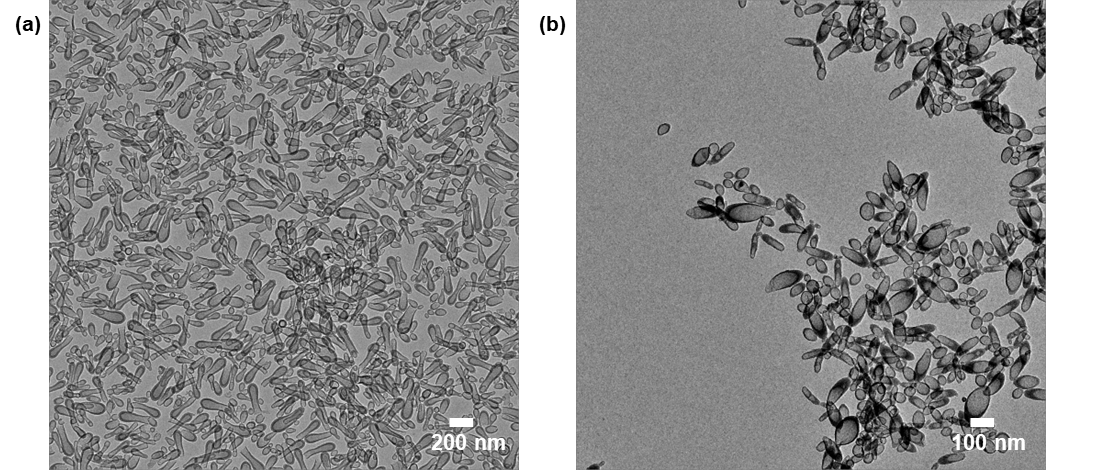


**Supplementary Figure 4.** Transmission electron microscopy (TEM) image of (a) PFCE@SiO_2_-C10-TAC (Scale bar: 200 nm) and (a) PFCE@SiO_2_-C8-TAC (Scale bar: 100 nm).


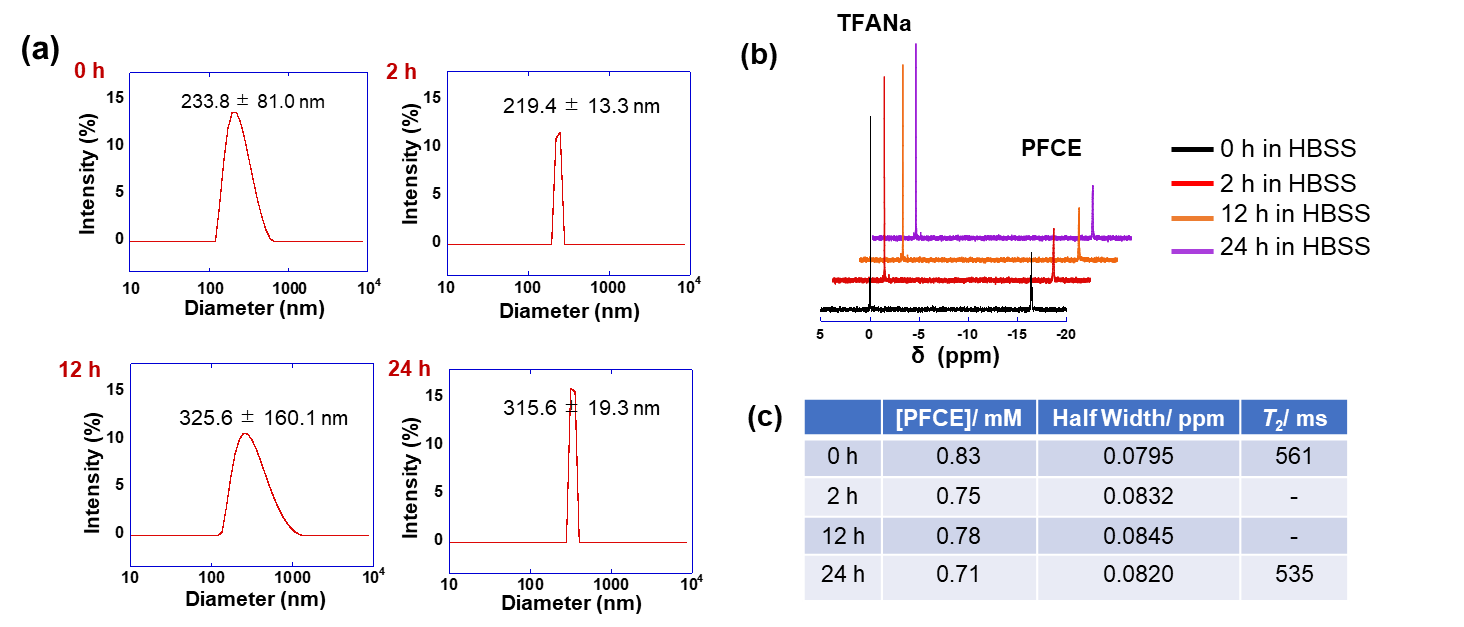


**Supplementary Figure 5.** Stability evaluation of PFCE@SiO_2_-C8-TAC in HBSS buffer. (a) Hydrodynamic radius of PFCE@SiO_2_-C8-TAC after 0, 2, 12, 24 h in HBSS buffer, respectively. Data measured by dynamic light scattering (DLS). (b) NMR spectra and (c) PFCE concentration and peak half width of PFCE@SiO_2_-C8-TAC after 0, 2,12, 24 h, and the *T*_2_ value of PFCE@SiO_2_-C8-TAC after 0 and 24 h in HBSS buffer, respectively.

**Supplementary Figure 6.** Cytotoxicity evaluation of PFCE@SiO_2_-C8-TAC in HeLa cells via WST assay (*n* = 3/ group).The cells were treated with PFCE@SiO_2_-C8-TAC with different concentration for 2 h.
